# The Ideals of Science

**Published:** 1892-07-09

**Authors:** 


### The Hospital

AN INSTITUTIONAL JOURNAL OF

### Science, Medicine, Nursing, and Philanthropy.

*For Hospitals, Asylums, Infirmaries, Dispensaries, Medical Practitioners, Students, Nurses, and the Charitable Public.*

VOL. XII.—No. 302.] [JULY 9, 1892.

### THE IDEALS OF SCIENCE.

AN Australian clergyman, whose contributions to theological literature are not inconsiderable, affirmed in a private letter the other day the somewhat disturbing proposition that the ideals of science are exceedingly "imperfect," not to say "poor." The present writer, as a humble lieutenant of science, felt somewhat taken aback. We are all more or less tinged with the religion of hero-worship; and some of us have hitherto thought that if there be a perfect goddess anywhere, pure science is the immaculate one. To many the question has never presented itself whether the ideals of science be "imperfect" or not. Casting one's eye in imagination over some of the workers in physiological, chemical, and other laboratories, one is inclined to doubt whether they have so much as thought out for themselves what the ideals of science might possibly be, or whether, indeed, science has anything at all to do with ideals. The average scientific worker excels as a craftsman rather than as a thinker. He is, indeed, inclined to despise thinkers. He puts his trust in his microscope and his eye rather than in his brain. Of course, such a worker is not, and never will be, a "man of science" in the proper sense of the words. He is a mere apprentice or journeyman in the scientific workshop.

No man in the world ever yet did anything considerable who was not an idealiser. If it be true that science is either without ideals, or has at best imperfect ones, her day is destined to be very brief. In order to get at the truth one has to compare men of science, and the objects they have in view, with other classes of men, and the objects they set before themselves. Taking scientific and other men in detail in this way, one perceives that the average scientist, like the average clergyman, merchant, poet, or journalist is an entirely common-place person. The differentiating touch of genius or distinction is not there. No amount of persevering labour will make the entirely common-place man distinguished or great. If we wish to get at the value of the ideals of science, as compared, say, with those of literature, or of religion, we must compare the outstanding and distinguished persons whose lives have been devoted to science with similar outstanding and distinguished persons whose lives have been devoted to literature or religion.

In a short article like the present we cannot begin at the beginning and extend our comparisons from the dawn of historic times. Science had small chance of showing what she was really made of until comparatively recent days. It would not be fair to compare the science of the first ten centuries of the Christian era with the literature or the theology of that period. Our comparison will be far more effective and convincing if we confine ourselves to recent times and to names with which all Englishmen are more or less familiar. Newton was a man of the modern period, so was Faraday, so too was Darwin, and so also are Tyndal and Huxley. These are not, on the whole, names that one writes down without some quickening of the intellectual pulse. There are those who would say that among the creators and inspirers of modern England and the world these five names ought to be mentioned. Every one of the five has been a man of ideals, and of similar ideals. Let us stand still a moment until we have assured ourselves what their leading ideals really have been and are.

If we may venture to speak for such outstanding and distinguished persons, we would say that their leading ideals have been two in number, and have been identical in each of the five men. They have all sought to the best of their ability to see and understand things as they are; and they have also —to the best of their ability—laboured to bring the truth within the comprehension of their fellowmen. Now, these ideals may in the first place be judged by themselves, and they may also have their value estimated by comparison with other ideals. To see things as they are: how many ideas has the world produced which have been more worthy or more fruitful than this? If any man desires to apply a rough and ready test of its value, let him ask himself how many of the men and women of his acquaintance do see things as they really are, or even make the attempt? Do we not, almost to a man, insist upon seeing things as we wish them to be; and are we not ready to quarrel and even to fight with every person who endeavours to make us see them otherwise?

The day of civilisation did not begin to dawn until some man of more intellect and courage than his neighbours saw certain things about him in a truer light than before, and strove to make his neighbours see them too. Such seeing and striving have often been the signals for hatred and persecution. They are so still, even among the most learned, the most cultured, and the most religious persons. Many a man who, seeing things more truly as they are, begins to tell his neighbours, finds such a hornet's nest about his ears that in pure astonishment and disgust he puts his tongue and his pen under lock and key for the rest of his life. Why, he argues, should I toil and labour to see deeply and truly, and to make the world see what I see, if it is such a fool as to suspect my motives, denounce my actions, and persecute me with venom and rage? The world may find out truth for itself, or do without it. Who could blame a man of science if he reasoned thus? But no men have had more to endure of this sort than some of those whose names we have mentioned. If Darwin had been a murderer, a parricide, or even a betrayer of his country, he would not probably have been denounced one hundredth part so much as he was denounced for merely striving to see things as they were, and to persuade his neighbours to use the same clear method of vision.

Was Darwin without ideals? Were his ideals imperfect or poor? His conclusions may have been wrong sometimes or even often; but his ideals were noble in the highest degree, and his achievements were almost equal to his ideals. A man does not need to be a Darwinian in order to see this; he needs but to have average intelligence, associated with fairness of mind.

But it is proper also to compare these ideals of the man of science with the corresponding ideals of other men—let us say, for example, with those of men of religion. What are the ideals of civilised religious men? Perhaps we may say the two leading ones are worship and amelioration: worship of the Divine and amelioration of the stern conditions of human life. And what is worship of the Divine but a deep and transforming admiration of the just, the true, the beautiful? No man of science will say one word against this condition of mind. On the contrary, if he be really a "man of science," and not a "journeyman or an apprentice," he will say that this is the very attitude of mind of all others which he himself most truly admires. He will say that it is this attitude, and this only, which makes him wish to see things exactly as they are, and to bring his fellow-men to his own state of mind. The seer of truth and the worshipper of perfectness belong to the same order of intelligence.

The religious man has, however, "amelioration" as one of his ideals; and it is here, from this standpoint, that he looks upon the ideals of the man of science as being imperfect and poor. Is there any justice in this judgment? Perhaps there is. Amelioration is a great ideal; it ennobles the mind of the ameliorator, and is almost infinitely advantageous to those whose lot is ameliorated. It is an idea which has constituted a leading principle in all great religions. The greatness of religions may be measured by the degree in which they are animated by the idea of amelioration. If the ideals of science fail at all in comparison with those of religion, they fail here. Is it impossible for science to become an ameliorator, and so to render her own ideals perfect? So far from its being impossible, the order of development leads us to anticipate that amelioration will be the one inspiring force which will give a new and living impulse to science. Religion had little of amelioration in it when it first took hold of the consciousness of man; and in the religions of the savage peoples of our own time amelioration is hardly known. As it was with religion, which was an early development of humanity, so it will be with the science which is characteristic of these later times. Knowledge, law, and truth come first; humanity, and brotherhood will follow in their train.

The modern world is for the many, not for the few. The many claim all the world for their own; the world not only of material things, but of science and literature and everything which is best. Governments, to-day, if they would survive must serve the many; and science too, if she is to continue to live in robust energy, can only live by being a servant and friend to men of every class and name. That which strives for betterment may live: that which strives for itself only must die.

